# In Memoriam

**Published:** 1883-05-20

**Authors:** T. S. Powell

**Affiliations:** Atlanta, Ga.


### *IN MEMORIAM.*

Dr. Thomas E. Marable died Auguat 1882. Memorial lines were never written for a nobler man and truer friend than are these in tender remembrance of Dr. Thomas E. Marable, of Peterburg, Virginia.

He was a class-mate of the writer at school. We afterwards read medicine at the same time and in the same village, though under different preceptors, and together attended lectures at the University of Pennsylvania in the city of Philadelphia. Being studious and faithful to his duties, the young medical student won deserved honor at his examination, and commenced to practice in his profession with a most encouraging prospect of success. But the timidity and sensitiveness of his gentle and sympathetic nature, made the "rough places" incident to his profession uncongenial with his tastes, and he soon abandoned the practice of medicine, and entered into the mercantile pursuits, in which he continued untii his death.

In early life Dr. Marable was united in marriage to the accomplished and charming daughter of Dr. Morrison, a distinguished physician of Lawrenceville, Virginia. A daughter, the fruit of this union, grew up with the beauty and amiability of both the father and mother, and was happily married in her girlhood to Mr. Dodson, of Petersburg. As she has no children, she and her widowed mother are the only members of hisfamily to mourn the death of a father who was themost tender and indulgent of parents, and a husband pre-eminent among the noblest and most devoted I have ever known. I feel that his loss is to this wife and daughter is irreparable; but there is always one soothing tender ray of light for those who weep at the removal by death of such a man as this, and that is, the undying memory of his love and gentleness, and all those noble, endearing qualities, the remembrance of which the heart to feel a sweet, though pathetic, thrill when it recalls the companionship of a loved one like him. Truly, not lost, but one "gone before."

I, who knew him so long and so well, never had a better and truer friend, nor a nobler companion, and one more truly beloved; and as I sit here to-night and pen these lines to his memory, my mind restrospects the many past years, and seems to take in at a glance all the beauty and symmetry of character that I found in him during our intimate friendship since we were careless, happy boys playing together among the beautiful hills of our dear old State of Virginia.

The subtle sophistries of philosphy, "charm they never so wisely," to steal away man's hope of immortality, can never offer the faintest compensation for the happy, child-like faith that pierces the dark mazel of the unknown and sees the glory of the soul's eternal Shekinah; so, our supersition will not be fo ever. O, my cherished, life-long friend, the glad hope of meeting you one day on the "other shore," where earthly love is renewed in never-to-be-broken bonds, will cheer the remaining pathway here of wife and daughter, and he who pens these lines will hold your name and your friendship in everlasting remembrance.

*Atlanta, Ga.* T. S. POWELL.

---

DR. DARBY, of Morrow, Ohio, proposes a novel plan of instructing the people how to treat doctors. He publishes what he styles "Medical Miscellany," which " is a double columned, four-paged sheet, designed to be used as powder and wrapping paper by dispensing druggists and physicians. It may be torn or cut into halves, quarters, eighths, or sixteenths, without destroying the reading matter.

This sheet is compiled wholly in the interest of the medical profession. Its teaching embodies that article of the Code of Ethics which relates to "the duties of patients to their physicians," a portion of which will be found in short paragraphs, conspicuously displayed, in large type, on one or both sides of each sixteenth of a sheet, interspersed among useful receipts and items of wit and wisdom, so as to interest the general reader.

Some method of thus quietly educating the public in their duty toward us as a profession has long been felt, and this one must strike you as at once new, novel and effective." Dr. Darby's plan is a good one, and we heartily approve it.

